# Dental caries status of Dai preschool children in Yunnan Province, China

**DOI:** 10.1186/1472-6831-13-68

**Published:** 2013-11-27

**Authors:** Shinan Zhang, Juan Liu, Edward CM Lo, Chun-Hung Chu

**Affiliations:** 1 Faculty of Dentistry, The University of Hong Kong, Hong Kong SAR, China; 2 School of Stomatology, Kunming Medical University, Kunming, Yunnan Province, China

**Keywords:** Caries, Children, Ethnic, Minority, China

## Abstract

**Background:**

The Dai people, one of the ethnic minorities in China, have a population of 1,260,000. They have the same origin as one of the main ethnic groups of Laos and Thailand. Most of the Dai live in Yunnan province, which is located in the less-developed southwestern part of China. This study aimed to describe the oral health status of Dai preschool children in China and the factors that influence their oral health status.

**Methods:**

An oral health survey was performed between 2011 and 2012 to select Dai five-year-old children using multi-stage stratified sampling in Yunnan. Their dental caries experience was measured using the “dmft” index, and severe caries was assessed using the “pa” index, which is modified from the “pufa” index. Oral hygiene status was assessed using the visual plaque index (VPI). A questionnaire to study the children’s socio-demographic background and oral health-related behaviours was completed by the children’s parents.

**Results:**

A total of 833 children were examined. Their caries prevalence was 89% and 49% of the children had carious tooth with pulp involvement. The mean (SD) dmft score was 7.0 (5.3). Higher dmft scores were found among children who were girls, were currently bottle-fed, took daily sweet snacks, had higher VPI scores, and had visited a dentist within the last year.

**Conclusions:**

The caries prevalence and experience of the five-year-old Dai children in Yunnan, China was high, and almost half had severe caries. The caries experience was associated with gender, snack habits, dental visit habits, and oral hygiene status.

## Background

China is the second largest country in Asia according to geographical area, and it has 56 ethnic groups. Han, the predominant ethnic group, constitutes approximately 92% of the total population of 1.351 billion in 2012 [1]. The other 55 ethnic groups account for only 8% of the total population of China. However, their population is approximately 114 million [1]. They are widely scattered across the country, but the majority live in inland or border districts in the less-developed western regions. These ethnic minorities are culturally and linguistically diverse. They speak over 80 languages, of which 30 have distinct written forms [2]. To deal with the large number of cultures, languages, and social statuses, the central government has set up a regional ethnic autonomy system. Earmarked funds and material support are provided, and bilingual education policy (Mandarin Chinese or Putonghua and ethnic dialect) is carried out, aiming for a holistic development of the ethnic minority groups. However, there is little health policy formulated to cater to their needs, and dental health is a field that is neglected.

Studies in different countries have reported that minority ethnic groups often live in disadvantaged communities and shoulder a disproportionate burden of oral disease [3,4]. One literature review concluded that socio-economic status is one of the most important factors in inequality [4]. Moreover, underlying cultural customs and beliefs can influence oral health status through diet, dental care-seeking behaviour, or the use of home remedies [5]. These factors are fundamental in framing appropriate oral health policies and the development of effective oral health services and oral health promotion activities [6]. In China here have been three national oral health surveys, which were performed in 1983, 1995 and 2005. Ethnicity of the surveyed participants was not a fundamental category for data collection in the national oral health surveys. There is no information on prevalence and severity of dental disease among, dental service utilization by, or treatment provided to the ethnic minority patients.

There are 20 ethnic minorities in China that have populations larger than 1 million [1]. Dai is one of them, with a population of about 1,260,000 [7]. In China, most of the Dai people (97%) live in the southwest part of the subtropical valley areas of Yunnan province. Yunnan Province is situated in the far southwest part of Mainland China. As a frontier province, Yunnan shares borders with three countries: Myanmar on the west and Laos and Vietnam on the south. It is also connected to Thailand and Cambodia by the Mekong River waterway. The province has an area of 394,000 square km. Some Dai people live near the Mekong River where it meanders through the far south of Yunnan. Dai is one of the main ethnic groups in Laos and Thailand, and Dai people also live in other Asia countries, such as Cambodia.

The history of the Dai people in China can be traced back to 109 BCE, when they established their own country [8]. They have their own language, which belongs to the Chinese–Tibetan language family and is written in an alphabetic script. The majority are Buddhist. Most (91%) Dai people in Yunnan live in rural areas, and about 81% are engaged in farming. The Dai people are typically farmers, growing a variety of tropical crops, including rice. In 2010, the gross domestic product (GDP) per capita of the Dai autonomous prefectures was around US$ 2,200, which is 84% and 46% of the provincial and national GDP per capita, respectively [9,10].

Dai people have their own special oral health attitudes, behaviours, and habits that can be dated back to the Tang Dynasty (618–907 CE). It was recorded that the men of Dai decorate their teeth with gold or silver as a symbol of elegance [8] and that the women painted their teeth with a thick layer of black soot, which was considered a symbol of elegance and thought to prevent tooth decay [11]. These habits or beliefs may have significant impact on the Dai people’s current oral health status.

Although the use of fluorides and advances in caries prevention have a significant impact on dental caries, many children, particularly in disadvantaged communities, still suffer from early childhood caries. The World Federation of Public Health Associations (WFPHA) made a declaration during the WFPHA 2013 General Assembly that oral health problems in children can impact many aspects of their general health and development, causing substantial pain and disruption to their lives and often altering their behaviour. Oral health is an integral part of overall well-being and is essential for eating, growth, speech, social development, learning capacity, and quality of life [12]. Every child has a right to good oral health. However, epidemiological data on the caries status among Dai ethnic minority children are scarce. Two studies using convenience samples showed that the Dai preschool children had a higher prevalence of caries than that of other local ethnic minority children [13,14]. The studies did not investigate social determinants of the children’s dental health.

This present study was performed to investigate the caries status of Dai children in China. The aims of the study were first to assess the caries and severe caries status and second to investigate the risk factors for caries in five-year-old Dai children in Yunnan, China.

## Methods

### Selection of children and sample size

This study was performed between 2011 and 2012 with ethics approval of the Institutional Review Board of the University of Hong Kong (IRB UW-11-377). A multi-stage, stratified sampling method was used in child recruitment. The recruitment of children was performed with the help of the Bureau of Public Health and Bureau of Education of the local government. Yunnan has sixteen districts. According to the population distribution of Dai people in Yunnan Province [8], most live the southern and the western districts. In the first stage, a southern district (Xishuangbanna) and a western district (Lincang) were chosen which have the largest concentration of Dai people. In the second stage, two counties of Xishuangbanna and three counties of Lincang with the highest population of Dai were selected. In each county, all kindergartens were invited to participate in this study. All five-year-old Dai children in the selected kindergartens were invited to participate in this study. Written informed consent for participation in the study was obtained from their parents. Children with major systemic diseases or syndromes, or on long-term medication, were excluded from the study.

A pilot study using a convenient sample of 127 five-year-old children in Lincang, Yunnan was performed, and the caries prevalence was found to be 81% [15]. A confidence interval of 78% to 84% was set (the standard error was 1.5%). The sample size required in this survey was 684. The response rate was estimated to be 85% and thus this study aimed to recruit 805 children.

### Questionnaire survey

A visit was paid to each kindergarten before the survey to deliver the questionnaire and discuss the protocol. Parents were asked to complete a questionnaire (attached as Additional file 1) that included (i) demographic information (age and gender of the child and education level of parents); (ii) oral health-related behaviours (the child’s snacking habits, tooth brushing practice, and dental visit), and (iii) perceived oral health of the child.

### Clinical examination

The clinical examination of the preschool children was conducted by a trained and calibrated public health dentist in the kindergarten using a ball-ended CPI probe and a disposable dental mirror attached to an intra-oral LED light. Dental caries status was accessed using criteria recommended by the World Health Organization [16]. Caries is diagnosed when a lesion has an unmistakable cavity, undermined enamel, or a detectably softened floor or wall. The dmft index was used to record the caries experience of the primary dentition: “d” stands for a decayed tooth, “m” denotes a missing tooth due to decay, and “f” represents a filled tooth.

This study used an index to record the severely untreated decayed teeth with pulp involvement and abscess or fistula—the “pa” index, which is modified from the “pufa” index [17]. The “p” denotes an untreated carious tooth with visible pulp involvement, and “a” denotes an untreated carious tooth with visible apical infection that can be in the form of a fistula or abscess. Oral hygiene status was measured using the visible plaque index (VPI) [18]. It involves the visual recording of the presence or absence of dental plaque on the buccal surfaces of six index primary teeth (55, 53, 51, 71, 73, and 75). The percentage of the six examined sites with visible plaque was calculated, and ranged from 0% to 100%. Duplication examinations were performed on 10% of the children. The Kappa statistic was used to assess the intra-examiner reproducibility.

### Data entry and analysis

The data collected were analysed using SPSS 20.0 software. Independent t-test and one-way ANOVA were used to assess the statistical significance of the differences in dental caries experience (mean dmft scores) between groups. Multiple comparison using the Bonferroni test was performed to compare the groups (N > 2) when the independent variable was found to be a significant factor affecting the caries experience (mean dmft scores). Multi-factor ANCOVA was used to investigate the effects of the independent variables studied, including the demographic background of the child and the child’s oral health-related behaviours and oral hygiene status on the child’s dental caries experience (dmft scores). The dependent variable was the child’s mean dmft scores, and all the independent variables were entered into the model. The statistical significance level for all tests was set at 5%.

## Results

A total of 891 Dai children from 29 kindergartens were invited; 833 attended the clinical examination. The response rate was 95% (833/891). Among those who did not attend the clinical examination, 5 (0.1%) declined to participate and 53 (5%) were absent on the day of examination. There were 406 boys and 427 girls who participated in this study. Their mean (SD) age was 5.3 (0.7) years. The Kappa’s values for scoring dmft, pa and VPI were 0.98, 0.94 and 0.89, respectively.

Table 1 shows the caries experience, caries prevalence, clinical consequences of untreated dental caries, and their prevalence amongst the Dai children. The caries experience in mean (SD) dmft was 7.0 (5.3). Most of the caries found was left untreated (dt = 6.8, 97%). The majority of the children (89%, N = 741) had caries experience, and only 11% (N = 92) had no caries experience (dmft = 0). There were 67% (N = 558) of the children with a dmft score of greater than 4, and 27% (N = 225) had a dmft score of 10 or above. For those children who had untreated caries (dt > 0), 56% (N = 408) had at least one tooth with severe caries (pa > 0) and 20% (N = 150) had visible apical infection in form of fistula or abscess.

**Table 1:** Caries experience, caries prevalence and clinical consequence of untreated caries of Dai children (N = 833)

**Dental caries**	**Prevalence**	**Mean (SD)**
Caries experience (dmft)	89%	7.0 (5.3)
Decay teeth (dt)	88%	6.8 (5.2)
Missing teeth due to caries (mt)	9%	0.1 (0.5)
Filled teeth (ft)	3%	0.1 (0.4)
Prevalence of severe caries – pulp involved (pa)	49%	2.1 (3.3)
Visible apical infection – abscess or fistula (a)	18%	0.3 (0.7)

**Figure 1:**
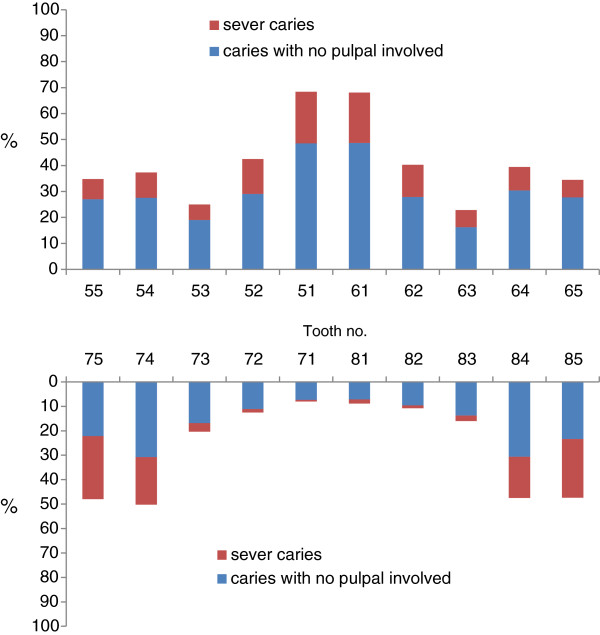
Untreated caries prevalence according to the tooth.

The prevalence of severe caries teeth was high among lower second molars (52%), lower first molars (37%) and upper central incisors (31%) (Figure 1). Untreated dental caries had a higher risk on the mandibular teeth than on the maxillary counterpart, and on the posterior teeth than the anterior counterpart. There were 76% of children with caries in their maxillary anterior teeth, and this was found to be associated with the prevalence of caries in their posterior teeth (p < 0.001).

There were 21% (N = 176) of the children who had no visible plaque detached on their teeth, whereas 6% (N = 53) of the children had visible plaque detected on all their index teeth. The mean (±SD) VPI score was 48% ± 30%. Higher VPI scores were found among the children who did not or seldom brushed their teeth than among the children who had daily tooth brushing habits (60% ± 29% vs. 45% ± 29%; p < 0.001).

Caries experience according to variables studied is shown in Table 2. Higher dmft scores were found in the Dai five-year-old children who lived in villages, were currently bottle fed before sleep, had snacks daily, had no tooth brushing daily, had visited a dentist before, and whose parents did not have tertiary education. Multiple regression analysis showed that higher dmft scores were found among the girls who were bottle fed, had daily snacking habits, had visited a dentist within the last year, and had a higher visible plaque score (Table 3). Among the children with untreated caries, multiple regression analysis (adjusted R^2^ = 0.340) found that there were more severe caries in children who were bottle fed before sleep (p < 0.001), had visited a dentist before (p < 0.001), and had a higher visible plaque score (p < 0.001).

**Table 2:** Caries experience (dmft) and variables studied

**Variables (%, N)**	**dmft (SD)**	**p-value**
Gender		
Boys (49%, 388)	6.8 (5.3)	0.387
Girls (51%, 398)	7.1 (5.3)	
Location		
Towns (49%, 388)	5.8 (5.0)	<0.001
Villages (51%, 398)	8.2 (5.3)	
Father’s education		
Secondary or below (84%, 660)	7.4 (5.3)	<0.001
Tertiary or above (16%, 125)	4.9 (5.0)	
Mather’s education		
Secondary (87%, 673)	7.3 (4.6)	<0.001
Tertiary or above (13%, 104)	4.6 (4.6)	
Parent take care		
Yes (77%, 601)	6.8 (5.3)	0.182
No (23%,181)	7.5 (5.3)	
Bottle feeding before sleep		
Yes (19%, 154)	8.1 (5.3)	0.003
No (81%, 626)	6.7 (5.3)	
Daily snacking		
Yes (64%, 502)	7.6 (5.3)	<0.001
No (36%, 282)	5.9 (5.1)	
Daily tooth brushing		
Yes (76%, 590)	6.7 (5.1)	0.002
No (24%, 189)	8.1 (5.6)	
Visited a dentist		
Yes (26%, 202)	9.2 (5.3)	<0.001
No (74%, 583)	6.2 (5.0)	

**Table 3:** Relationship between dental caries experience and the selected independent variables (final model of multi-factor ANCOVA) (N = 743)

**Independent variables**	**Group**	**Beta**	**SE**	**p-value**
Gender	Boys	−0.849	0.247	0.001
	Girls^a^			
Father education	Secondary or below	0.728	0.347	0.036
	Tertiary or above^a^			
Bottle feeding before sleep	Yes	0.995	0.310	0.001
	No^a^			
Daily snacking	Yes	0.667	0.260	0.011
	No^a^			
Visited a dentist	Yes	1.681	0.288	<0.001
	No^a^			
VPI		12.400	0.429	<0.001
Intercept		−0.322	0.384	0.402

^a^Reference Category, Adjusted R^2^ = 0.586.

## Discussion

Since the majority of the Dai people in China are living in Yunnan province and are scattered in rural areas, this study used a multi-stage and cluster sampling method to include the Dai preschool children. It is important to note that attending kindergarten is not compulsory for preschool children. Some children living in rural areas might not go to kindergarten and were not able to be included in this survey. Although the number should be small, it could affect the prevalence and caries experience of Dai children. Thus, the results of this study should be interpreted with caution. Multi-stage sampling is generally considered a more accurate cluster sampling for the sample size. In this multi-level cluster sampling, representative clusters are chosen and every child within the chosen cluster is sampled. It is a convenient and effective method of finding the survey sample and is particularly suitable for this study, which requires a large sample size. It is noteworthy that children selected using the multi-stage cluster sampling method may not be as representative as those selected through random sampling. However, random sampling can be difficult to perform in a survey, particularly when people are scattered over large and remote areas. The diverse geographical settlement of Dai people makes random sampling very costly and slow to carry out. This method is also useful when a complete list of all children of the population does not exist or cannot be obtained.

In this study, we recruited a large sample size and a high response rate of five-year-old children. The support from the local government and the good rapport developed through pre-survey visits to kindergartens were probably two major reasons for the satisfactory outcome. The pre-survey visit also allowed the kindergarteners to understand the aim and method of the survey. Invitation letter, consent forms, and questionnaires were also delivered to parents through the kindergartens with sufficient time provided for collection.

Caries and severe caries have a considerable impact on preschool children’s quality of life [19]. Monse et al. proposed the “pufa” index to assess clinical consequences of untreated dental caries [17]. The pufa index is an epidemiological tool complementary to existing caries indices aimed at assessing dental caries [20]. Figueiredo et al. (2011) suggested that code “u” can be deleted and codes “f” and “a” can be combined in the assessment [20]. This study therefore assessed the severe caries with “pa” index. The examiner in this study found the “pa” index to be simpler than the “pufa” index, and a high Kappa value was obtained in this study.

Most Dai people live in the subtropical zone, where sugar is produced. The availability of sugar promotes its consumption in various forms, such as sweets and soft drinks. Sticky rice is one of their main foods, and some parents use sugar cane as a pacifier to calm crying children, which can accelerate the accumulation of plaque and prolong the time of acid production. The caries experience measured by mean dmft of the surveyed Dai children was nearly double that reported by the National Committee of Oral Health in 2008 [21]. The increase in caries experience is alarming, and oral health promotion is crucial for prevention of dental caries. There is no organized caries prevention program for children living in rural areas in China [22]. This study found high caries experience among the children living in villages. It is plausible that children in villages had unsatisfactory oral hygiene practices, frequent snacking, and lack of easy access to dental care. In addition, tooth brush and fluoride toothpastes can be ‘expensive” to be used by the children. There are also no oral health education materials in the Dai language. The low education level of parents and the language barrier may be another reason for the severe caries status in villages.

Dai is a major ethnic group in Thailand. The caries prevalence in this survey is similar to that of the five- to six-year-old Dai children in Thailand [23]. The similarity in ethnic origin, culture and lifestyle may be one of the reasons for these findings. The caries experience of the Dai children in China was lower than that of the children in rural Cambodia [24]. This difference may be related to social culture and geographical differences of the two study groups in China and Cambodia. The dentist to population ratio is even smaller in Cambodia that than in China. Unlike China, Cambodia does not have assistant dentists to serve the community [24]. In addition, there may have different oral health related belief, dental care-seeking behaviour, and availability of clean water supply. Caries was more prevalent in mandibular molars and maxillary incisors among the Dai children in this study. This distribution of caries is similar to a study on preschool children [25].

In this study, Dai boys had a lower mean dmft scores than girls, who had higher caries experience. This finding is different from that reported in the last two national oral health surveys, which found no significant difference of caries experience between boys and girls [21,26]. This study also found no gender difference in tooth brushing habits or dental visit behaviours. Gender discrimination against female children still exists in some Dai ethnic minority families, and it is not known if this could contribute to this difference in the caries experience.

Although dental caries is caused by plaque on the tooth surface, strong clinical evidence showing that tooth brushing per se is effective in caries prevention is lacking [27]. The Dai children in this study, however, experienced fewer caries when they practiced daily tooth brushing. This finding is in agreement with a number of earlier studies in various child populations [24,28]. It is noteworthy that analysis demonstrated that tooth brushing was not a confounder and did not remain in the final multivariate model, showing that its influence on the caries experience of the study children was not strong. This might be because many preschool children brushed their teeth with little support from their parents and their brushing was not very effective due to their young age. A higher caries experience was found in the children who visited a dentist. This finding was also found in a previous study [29]. Problem-oriented dental care-seeking behaviour is common in China. It is plausible that children were brought to visit dentists for pain and infection. Effort should be made to promote early and regular preventive dental visits among young children to change this pattern of dental care-seeking behaviour. The establishment of a public or a subsidized prevention-oriented dental service for preschool children would probably help to improve the situation.

## Conclusion

In this survey, the caries prevalence and experience of 5-year-old Dai children in Yunnan, China was high. For those children who had untreated caries, more than half had severe caries and one fifth had visible apical infection in the form of a fistula or abscess. The caries experience was associated with gender, snack habits, dental visit habits, and oral hygiene status.

## Competing interests

The authors declare that they have no competing interests.

## Authors’ contributions

SZ is the main person to plan, perform the survey, and prepare the manuscript. The other three authors contributed equally to the supervision of this work, and read and approved the final manuscript.

## Authors’ information

Dr. Shinan Zhang is a PhD student in the Faculty of Dentistry, Prof ECM Lo, Dr. J Liu and CH Chu are supervisors of Dr. Zhang.

## Pre-publication history

The pre-publication history for this paper can be accessed here:

http://www.biomedcentral.com/1472-6831/13/68/prepub

## Supplementary Material

Additional file 1 Questionnaire. Click here for file
