# Supplementary material for: Dental caries status of Dai preschool children in Yunnan Province, China
Source: BMC Oral Health. 2013 Nov 27;13:68. doi: 10.1186/1472-6831-13-68 (PMC4222259; doi:10.1186/1472-6831-13-68)
Supplement: Additional file 1 — Questionnaire. [file 1472-6831-13-68-S1.pdf]

Case No: __________

Oral health survey of Dai children in Yunnan

1. Child name： _________________ Class： _________________

2. Contact number： ___________________

3. Child gender： □1 Male □2 Female

4. Where does the child live?

□1 Town □2 Village

5. Are parents the main care taker of the child?

□1 Yes □2 No

6. Is your child still sleep with a bottle with milk/sweet drink?

□1 Yes □2 No

7. Does your child brush his/her teeth daily?

□1 Yes □2 No

8. Does your child take snacks daily?

□1 Yes □2 No

9. Did your child visit a dentist in the last 12 months?

□1 Yes □2 No

10. Father's education level：

□1 Secondary or below □2 Tertiary or above

11. Mother's education level：

□1 Secondary or below □2 Tertiary or above

~ The End ~
